# Preparation of a Microwave-Absorbing UV Coating Using a BaFe_12_O_19_-Polypyrrole Nanocomposite Filler

**DOI:** 10.3390/polym15081839

**Published:** 2023-04-11

**Authors:** Ting Lai, Wenzhen Qin, Caogui Cao, Rong Zhong, Yun Ling, Yu Xie

**Affiliations:** Department of Materials and Chemistry, School of Environmental & Chemical Engineering, Nanchang Hangkong University, Nanchang 330063, China; laiting@163.com (T.L.);

**Keywords:** BaFe_12_O_19_, polypyrrole, in situ chemical polymerisation, UV curing coating

## Abstract

BaFe_12_O_19_-polypyrrolenanocomposites were prepared via the in situ chemical oxidative polymerization of pyrrole monomers in the presence of BaFe_12_O_19_ powder, with ammonium persulfate as an oxidant and sodium dodecyl benzene sulfonate as a dopant. X-ray diffraction measurements and Fourier-transform infrared spectroscopy indicated that there were no chemical interactions between BaFe_12_O_19_ and polypyrrole. In addition, scanning electron microscopy showed that the composites exhibited a core–shell structure. Subsequently, the prepared nanocomposite was used as a filler to prepare a coating suitable for ultraviolet curing. The performance of the coating was investigated by evaluating its hardness, adhesion, absorbance, and resistance to acids and alkalis. Importantly, the addition of BaFe_12_O_19_-polypyrrole nanocomposites not only improved the coating hardness and adhesion but also produced a coating with a good microwave absorption performance. The results suggested that BaFe_12_O_19_/PPy composite has a lower reflection loss peak and a larger effective bandwidth at the X band when the proportion of the absorbent sample is 5–7%, when the absorption performance is the best. The reflection loss is in the range of 8.88–10.92 GHz below −10 dB.

## 1. Introduction

In recent years, electromagnetic-wave-absorbing materials that are thin, lightweight, and that exhibit wide and strong absorption have received growing attention. More specifically, electromagnetic-wave-absorbing materials based on conductive polymers have been reported to exhibit a range of desirable characteristics, including a low specific gravity, adjustable electromagnetic parameters, good compatibility, and low cost, rendering them a key research topic in the area of microwave-absorbing materials [1,2,3,4,5]. To date, the conductive polymer materials that have received the most attention are polyacetylene, polyaniline, polypyrrole (PPy), and polythiophene, whose microwave absorption properties can be attributed to their large, conjugated bond systems that promote electrical conduction. Although absorbing materials composed of conductive polymers are known to exhibit dielectric loss, the magnetic loss is largely unaffected. As a result, doping with an appropriate filler is necessary to achieve magnetic loss. Fillers can be divided into various categories, including metal-type conductive polymers, non-metal-type conductive ionizing molecules, oxides, magnetic powder-type conductive polymers, and doped conductive polymers, all of which exhibit absorption properties. Although inorganic microwave absorbents have developed quickly, they are also limited due to their high density and poor reprocessing performance. As a result, conductive polymers have emerged as a new generation of wave absorbing materials. Conductive polymers have several excellent properties compared to inorganic microwave absorbers: First, their density is low, generally not greater than 2.0 g/cm^3^. The use of this material allows for flexible movement and light weight. Secondly, conductive polymers have a wide range of applications. The conductivity of the polymers varies widely between semiconductors, insulators, and conductors (10^−9^ to 10^3^ S/cm), and different conductivity values exhibit different wave absorbing capabilities, which makes it possible to design various conductive polymers according to different needs. Thirdly, conductive polymers have quite good thermal stability and can better adapt to the environment.

Metal-type conductive polymer fillers generally contain iron powder, nickel, and manganese. Among the numerous studies performed in this field, Anil Ohlan et al. [6] synthesized nano barium ferrite doped poly (3,4-dioxythiophene) shell core composite polymer (PEDOT BaF) through in-situ lotion polymerization. The polymer was analyzed by Fourier transform infrared spectroscopy (FTIR), XRD, energy dispersive X-ray spectroscopy (EDS), and high-resolution transmission electron microscopy (HRTEM). Magnetic losses such as hysteresis, domain wall displacement, and eddy current loss of ferrite nano ions in the polymer were obtained. An increase in ferrite composition was observed at 22.5 dB μ = 0.22 magnetic loss and ξ = 23.5 conductivity, which contributes to microwave absorption. Lee et al. [7] prepared absorbing materials based on a PPy and polyester fiber composite, and they found that the addition of silver powder and palladium powder increased the absorption capacity to 55 dB. Non-metallic-type fillers mainly consist of carbon black, carbon fiber, or graphite. For example, Biscro et al. [8] reported a resin composite absorbing material filled with T300 chopped carbon fiber and found that the reflectivity of this material was <10 dB in the frequency range of 8–12 GHz. In addition, Luiza et al. [9] prepared a carbon fiber–polyaniline composite whose incident-electromagnetic-wave-absorption efficiency could reach 87%. The main oxide and magnetic-powder-type conductive polymer materials used as fillers were titanium oxide and zinc oxide. Similarly, Feng et al. [10] described a composite containing an iron-based powder and polypropylene vinyl resin, and they found that the reflectivity of a 3-mm-thick coating of this material at 3.5 GHz was −25 dB. Furthermore, a report from the University of Pennsylvania demonstrated that a 2 mm film composed of polyacetylene exhibits a microwave absorption capability of 90% at 5 GHz. Additionally, Olmedo of France studied the microwave absorption performances of PPy, polyaniline, and poly-3-octylthiophene in the range of 0–20 GHz, obtaining an average attenuation of 8 Db for poly-3-octylthiophene, along with a maximum attenuation of 36.5 Db and a bandwidth of 3.0 GHz.

Currently, the majority of traditional absorbing coatings are attached to the desired material by thermal curing. Radiation curing technology began in the 1960s, including electron beam curing (EB) and ultraviolet curing. Radiation curing refers to a new technology in which liquid oligomers (including monomers) undergo cross-linking polymerization after irradiation to form solid products. UV curing technology has “5E” characteristics: efficient, easily adaptable, economic, energy saving, and environmentally friendly. In the past decade, with the continuous development of UV curing technology, it has been widely used in coatings, microelectronics, ink, adhesives, biomaterials, automobiles, and other fields.More specifically, ultraviolet (UV) curing systems are widely employed due to their low energy consumption and their ability to provide stiff, strong, and highly adhesive materials that solidify rapidly [11,12,13]. In addition, this technology is recognized as being environmentally friendly because it releases only low quantities of volatile organic compounds [14]. However, there has been limited use of radiation curing technology for electromagnetic wave absorbing materials.

The research on spinel ferrite wave absorbers has a long history for scientists all over the world. However, due to the small anisotropic field H_a_, its magnetic permeability and absorption characteristics in the microwave frequency band are inferior to those of hexagonal ferrite. Hexagonal ferrite has a lamellar structure and high magnetic crystal anisotropy field Hκ and has a high white resonance frequency, fm, and good microwave absorption ability in the microwave section. The combination of spinel ferrite and hexagonal ferrite can effectively improve the electromagnetic performance of both, achieving good wave absorption effects in high and low frequency bands. Chen et al. [15] successfully synthesized SrFe_12_O_19_@ZnFe_2_O_4_. Their research results show that the composite effectively combines strontium ferrite and a ferrite-based core-shell structure. The composite powder is well coated, with a clear layered interface, and a shell thickness of about 5 nm. In the frequency range of 8 to 18 GHz, microwave absorption gradually increases. When the frequency is 12 GHz, the microwave absorption of the nanocomposite powder reaches a maximum value of −9.7 dB, making it an excellent microwave absorbing material.

BaFe_12_O_19_, a type of hexagonal ferrite magnetic material [16], is known to exhibit a large magnetocrystalline anisotropy, a high coercivity, and good magnetization properties. It has also been demonstrated to improve the conductivity of PPy [17]. More specifically, coating BaFe_12_O_19_ with PPy decreases the reflection loss of BaFe_12_O_19_ at 2–18 GHz [18,19]. When this doped nanoparticle species is added to a UV-curable system as a filler, the microwave absorption properties of the resulting film can be modulated simply by controlling the thickness of the film. However, compared with the traditional thermal curing process, the UV curing process has significant room for improvement [20,21,22,23,24]. Despite this, it has been reported that upon altering the composition of the coating species, the UV coating can adapt to a wide range of base materials, thereby exhibiting increased applicability [25,26,27,28]. In our previous work, it was found that doped nanoparticles are easily incorporated into UV-curable systems, although the filler distribution becomes uneven after curing. Moreover, to date, few studies have focused on the use of inorganic materials as UV-absorbing coatings.

Thus, we herein report the preparation and optimization of BaFe_12_O_19_-doped PPy. This composite is used to fill a UV-curing system as an absorbing agent and filler. Furthermore, a novel UV-absorbing coating is prepared to protect samples from environmental UV radiation. Quick and simple preparation of microwave absorption materials by UV curing technology is one of the aims in this study.

## 2. Experimental

### 2.1. Materials

Pyrrole, BaFe_12_O_19_, sodium dodecyl benzene sulfonate (SDBS), ammonium persulfate (APS), and three polyurethane acrylates (PUAs) (epoxy acrylate (EA), trihydroxymethyl propane triacrylate (TMPTA) and tripropylene glycol diacrylate (TPGDA)) were all purchased from Taiwan Changxing Chemical Co., Ltd. (Dongwan, Guangdong, China). Photoinitiator 1173 was purchased from Jiuri New Materials Co., Ltd. (Tianjin, China). All purchased reagents were of analytical grade and were used as received, without further purification.

### 2.2. Preparation of the Doped PPy

**Scheme 1:**
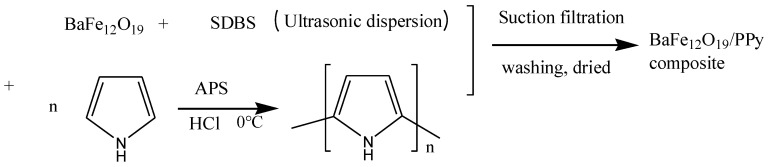
Preparation of thedoped PPy.

The doped PPy was prepared via two steps, as outlined in Scheme 1.

### 2.3. Synthesis of the BaFe_12_O_19_-PPy Nanocomposites

The BaFe_12_O_19_-PPy nanocomposites were synthesized via an in situ polymerization approach. An orthogonal test method was employed to determine the effects of APS and SDBS addition on the electrical conductivity of the composite. The same method was also employed to evaluate the influence of the reaction time and the ratio of components.

For preparation of the BaFe_12_O_19_-PPy nanocomposites, ferrite particles and SDBS were suspended in a 0.1 M HCl solution by ultrasonic dispersion over 1 h. After this time, pyrrole was added to the suspension and stirred for 30 min. The resulting solution was then maintained at 0–5 °C in an ice/water bath, and a solution of ammonium persulfate in HCl was slowly added dropwise to the suspension. Subsequently, the suspension was filtered and the residue was washed with absolute ethyl alcohol and deionisedwater several times. Finally, the target composite was obtained by drying under vacuum at 60 °C for 24 h.

### 2.4. Preparation of the UV Curing System

The composition of UV curing coating is listed in Table 1.

### 2.5. Characterization

The chemical structures of the obtained composites were evaluated using Fourier-transform infrared (FTIR) spectroscopy (Nicolet 6700, Thermo Fisher Scientific Inc., New York, NY, USA) with a resolution of 4 cm^−1^. The surface morphology of the material was examined using scanning electron microscopy (SEM, FEI Nova Nano SEM 200, USA) with a resolution of 1 nm and an accelerating voltage of 15 kV. The surface of the cured film was also subjected to X-ray diffraction (XRD) analysis (PANalytical Empyrean, The Netherlands) with a Cu Kα radiation X-ray source, a wide-angle scan range of 3–150°, and a goniometer accuracy of 0.0001°. For thermogravimetric/differential thermal analysis (TG/DTA, Perkin-Elmer Diamond, MA, USA), a small amount of film was scraped off the surface of the cured layer using a blade. Characterization was carried out in an argon atmosphere by heating from 25 to 800 °C at a rate of 10 °C/min. The light transmittance and UV absorption properties of the film coatings were tested using an LS160 transmittance meter (Shenzhen linshang technology Co., Ltd., Shenzhen, China).

The adhesion properties of the prepared coatings were evaluated with reference to the ASTMD3002 (0B-5B) standard. The test results are divided into 7 grades, namely 0–6, in which grade 0 refers to a coating film with a smooth edge and no peeling of the adhesive tape. The PET substrates used here were surface treated, and the paint film achieved grade 0 adhesion. The pencil hardness test was conducted according to the ASTM D3363-05 standard. A pencil was scratched five times on the film at a certain gravity (500 g or 750 g), and the hardness of the paint film was the pencil hardness that produced no scratch.

The scratch resistance of each specimen was evaluated as follows. Steel wool was used to abrade the vertical direction of the coating plane with a set number of passes at a fixed force. The haze of the sample before and after abrasion was recorded, and the change in haze was measured according to the haze change rate (ΔH/H_0_); a large change indicated poor scratch resistance. To evaluate sample flexibility, axis bars were marked as 1, 2, 3, 4, 5, 6, or 7, from large to small, according to their diameter, and the corresponding number was recorded for flexibility. It should be noted that a larger number indicates superior flexibility. To measure the resistance to acid, base and oil of the materials, a paint film was prepared on a glass slide and a tin plate. After the paint film had dried, 2/3 of the area was immersed in 93 # gasoline and silicone oil at a temperature of 25 ± 1°C, and 0.1 mol of hydrochloric acid and sodium hydroxide. After a certain time (6 h), the sample was taken out and dried with filter paper. The surface of the paint film was checked for wrinkles, blisters, peeling, discoloration, softening, and other phenomena.

## 3. Results and Discussion

### 3.1. FTIR Spectroscopy

**Figure 1:**
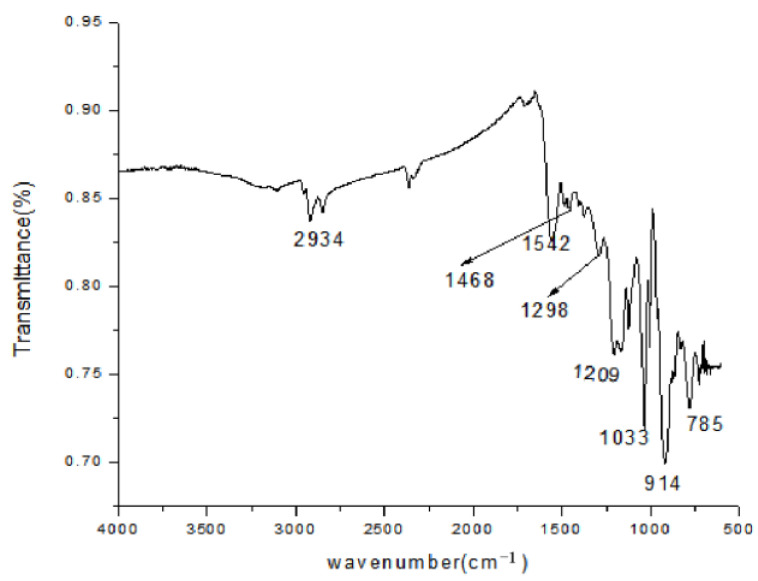
FTIR spectrum of PPy.

**Figure 2:**
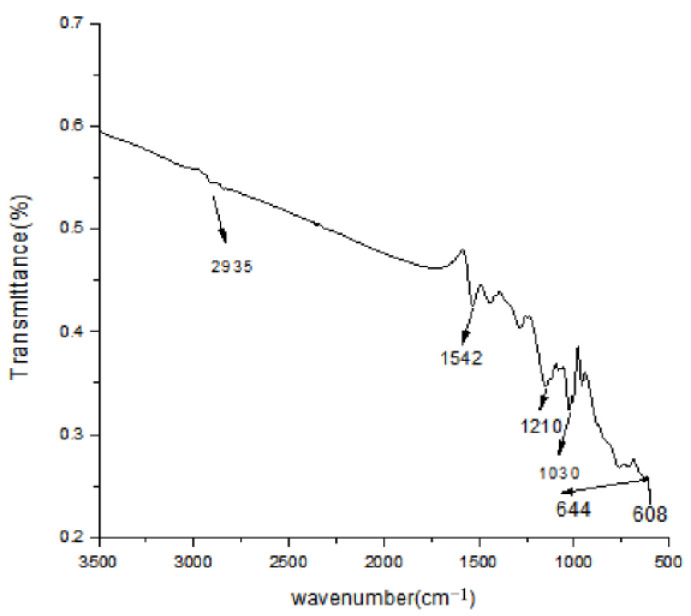
IR spectrum of the BaFe_12_O_19_-PPy composite.

Figure 1 and Figure 2 show the FTIR spectra of PPy and the BaFe_12_O_19_−PPy composite, respectively. More specifically, in Figure 1, the peak at 2934 cm^−1^ represents the characteristic N–H stretching vibration, while the peaks at 1542 and 1468 cm^−1^ correspond to the antisymmetric and symmetric stretching vibrations of the PPy ring, respectively. In addition, the peaks observed at 1298, 1209, and 1033 cm^−1^ originate from the in−plane deformation bending vibration band of the C–H moieties, while those at 914 and 785 cm^−1^ correspond to the out−of−plane bending vibration of the C−H groups [29]. In contrast, the adsorption peaks are slightly red−shifted in the composite because the PPy chains surround the ferrite particles, and as a result, the electron cloud density generated by their interaction concentrates the electron cloud originating from the polymer molecular chain. Thus, the combination of atomic vibration frequencies reduces the force constants between the constituent atoms, ultimately leading to a red shift of the absorption peaks. It should also be noted here that in the spectrum of the composite, two new weak peaks are observed at 644 and 608 cm^−i^, although no strong peak corresponding to the stretching vibration of the barium ferrite M–O bond is observed. These results further illustrate that the barium ferrite particles were coated by a polymer, thereby weakening the stretching vibrations of the M–O bonds.

### 3.2. X-ray Diffraction Measurements

**Figure 3:**
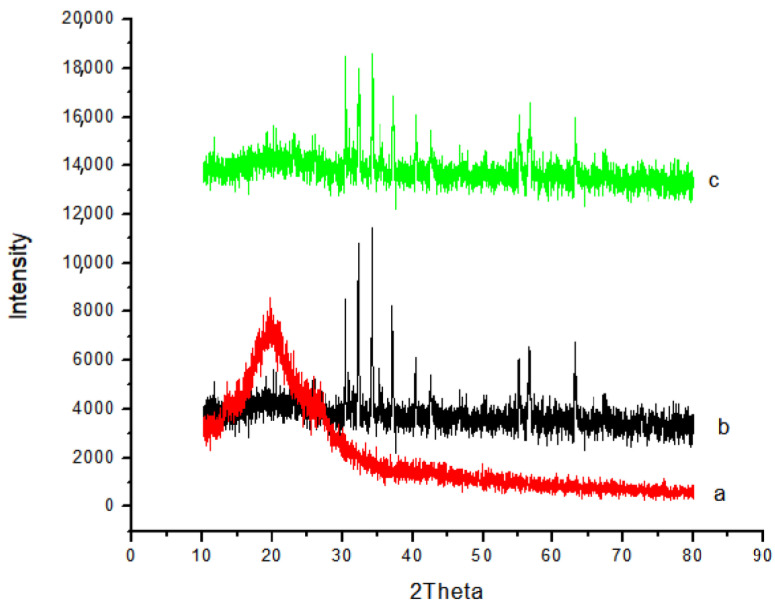
XRD patterns of (a) the pure PPy, (b) the pure BaFe_12_O_19_, and (c) the BaFe_12_O_19_-PPy composite.

Phase investigation was performed for the crystallized product PPy, BaFe_12_O_19_, and the BaFe_12_O_19_-PPy composite via XRD measurements, as shown in Figure 3. More specifically, the typical XRD pattern of PPy (Figure 3a) shows a broad diffraction peak centered at 2θ = 21°, which is a characteristic peak of this compound, although with a slight change in intensity and position compared to the spectrum reported in the literature [30]. In addition, Figure 3b shows the XRD pattern of the BaFe_12_O, while Figure 3c shows that of the composite, wherein a broad peak similar to that observed for PPy can be seen in addition to the characteristic ferrite peaks at 2θ values of 30.2, 32.6, 34.1, 37.2, 40.3, 55.1, 56.2, 63.1, and 72.3°. Upon comparing the patterns shown in Figure 3b,c, it is clear that the peaks corresponding to BaFe_12_O_19_ are weakened upon doping with PPy. This observation reveals that BaFe_12_O_19_ influences the regularity of the long PPy chains, ultimately affecting the crystallinity of PPy.

### 3.3. Morphology

**Figure 4:**
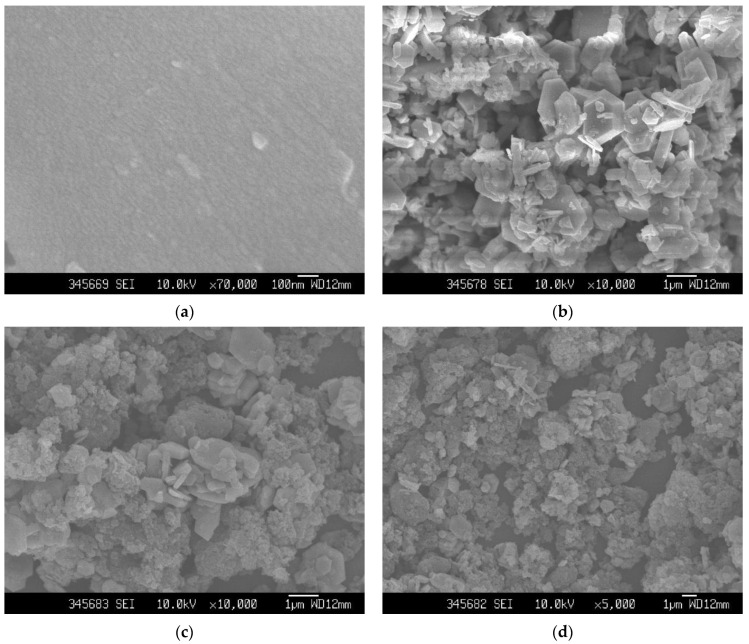
SEM images of (**a**) PPy and (**b**–**d**) BaFe_12_O_19_-PPy nanocomposite samples 1, 5, and 8.

The morphologies of PPy and the BaFe_12_O_19_-PPy nanocomposite were investigated by SME, as shown in Figure 4. Because APS is a water-soluble initiator, the polymerization reaction mainly occurs in the SDBS micelles. The reaction then proceeds at the emulsion/water interface, and as a result, micellar growth occurs along a fixed direction, forming a neat lamellar structure (Figure 4a). The particle size of barium ferrite is about 80 nm, showing a clear hexagonal lamellar structure, with a relatively uniform distribution. Some particles also have a small amount of agglomeration, which is due to the high surface energy of the nanoparticles, indicating that the hexagonal lamellar structure is an ideal shape as a nucleus.As APS is a water-soluble initiator, polymerization reactions mainly occur at the interface between the micelle and water after emulsification with SDBS. When the reaction proceeds further, the micelle will grow in a fixed direction to form a regular sheet like structure. It can be found that the growth of polypyrrole is carried out on the surface of barium ferrite, with a “climbing tiger” type of growth. When the ratio of BaF to Py is large, BaF is excessive in comparison to the insufficient Py monomers, resulting in a large amount of exposed BaF. When the proportion of polypyrrole is higher), polypyrrole can well coat most of the BaF (Figure 4c).

### 3.4. Coating Properties

The mechanical properties of the prepared coating are listed in Table 2, wherein it can be seen that complete UV curing of the composites is achieved rapidly within 1 min. In addition, the impact resistance of the coating is 48 cm and the adhesion to the substrate is level 1. Furthermore, the good acid, water, and oil resistances of the coating indicate that overall, the coating exhibited good mechanical properties.

### 3.5. Thermogravimetric Analysis

**Figure 5:**
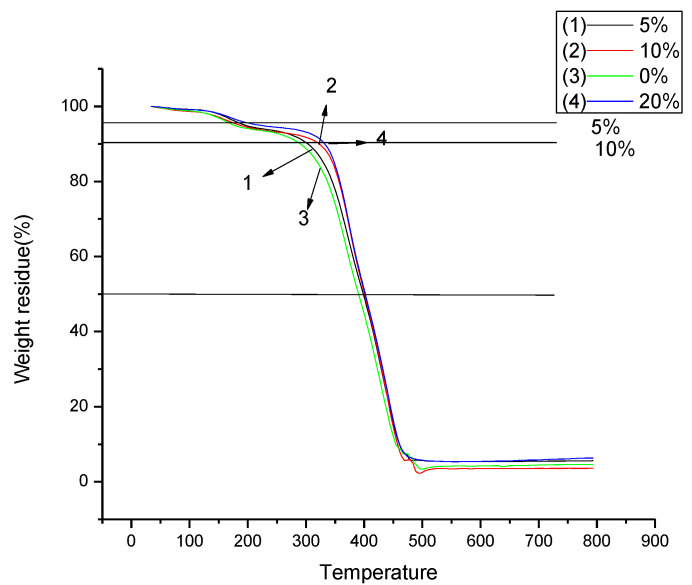
Effect of the absorbing agent loading on the thermal stability of the coating.

Figure 5 shows the TGA curves of the composites coated with 0, 5, 10, and 20% of the absorbing agent. During the sample heating process, a clear weight loss was observed between 330 and 480 °C for all samples, which was attributed to thermal decomposition upon bond fracture of the polymer curing system. In the absence of the absorbent, weight losses of 5, 10, and 50% were observed for the coatings at 163, 286, and 286 °C, respectively. However, with an absorbing agent content of 5%, the temperatures corresponding to these weight losses increased to 173, 298, and 298 °C, respectively. Similarly, with a 10% absorbing agent content, these temperatures increased further to 183, 322, and 322 °C, while at a 20% loading, they were 197, 332, and 332 °C, respectively. These TGA results show that upon increasing the amount of added absorbing agent, the thermal stability of the coating improved. This can be attributed to the fact that the absorbing agent possesses excellent thermal stability. Moreover, the incorporation of rigid nanoparticles into the organic phase produces a degree of steric hindrance, thereby limiting movement of the organic chains during heating.

### 3.6. Electrical Conductivity

The conductivity values of the prepared BaFe_12_O_19_-PPy nanocomposites were determined by means of orthogonal experiments and are presented in Table 3. From the obtained results, it is clear that the electrical conductivity is affected by the reaction time and by the amounts of added APS, SDBS, and BaFe_12_O_19_, wherein the latter has the greatest influence. As a result of optimization studies, the highest electrical conductivity was determined to be 6.971 S/m.

Conducting polymers are inherently conducting due to the presence of a conjugated π-electron system in their structure. The insulating behavior of the ferrite particles in the composite core hinders charge transfer, and the resulting generation of stable σ-π bonds between the PPy and the ferrite particles leads to a reduction in the conjugated π electron density [19].

**Figure 6:**
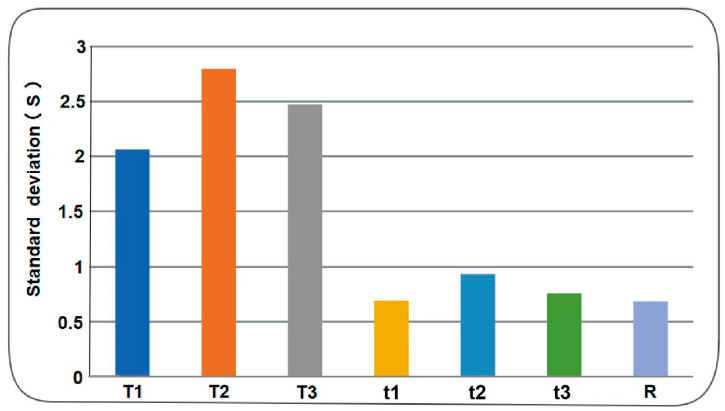
The standard deviation of the data analysis. (from left to right, T1, T2, T3, t1, t2, t3, R).

As outlined in Table 4, the factors responsible for influencing the polymer conductivity could be ordered as follows: APS content > BaF content > reaction time > emulsifier content. Upon subsequently comparing the average effect value of each factor at different levels, the order associated with the influence of the reaction time is T3 > T2 > T1, which indicates that the reaction time is conducive to improving the polymer conductivity. Similarly, the order associated with the influence of the APS loading is T2 > T3 > T1, indicating that a quantity of 2 g is required per mole of pyrrole. In the case of the emulsifier dosage, the corresponding order was determined to be T1 > T2 > T3, implying that the optimal emulsifier loading for 1 mole of pyrrole should not exceed 1.75 g. Moreover, for the influence of the BaF loading, the order was T3 > T2 > T1, indicating that lower quantities of BaF led to improved conductivity. The Figure 6 showed that the standard deviation of the data analysis. (T1, T2, T3, t1, t2, t3, R) It was therefore clear that the optimal conditions to obtain the maximum conductivity are A3B2C1D3.

### 3.7. Reflection Loss Tests

**Figure 7:**
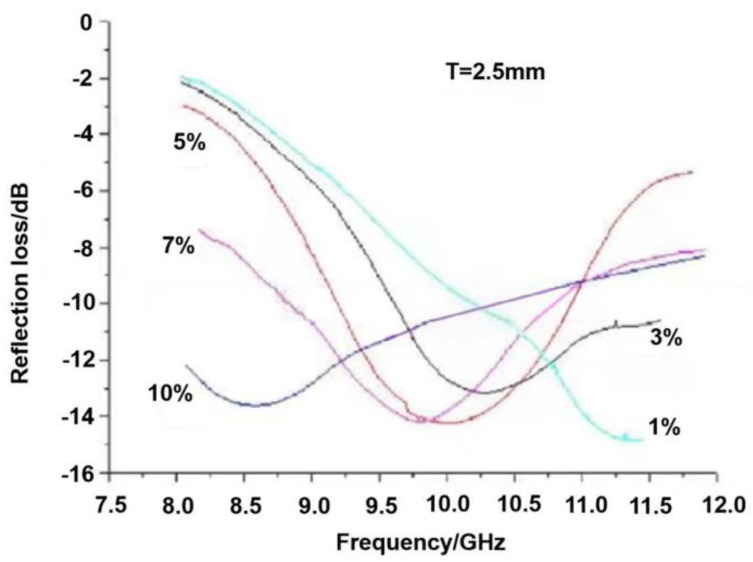
Influence of the absorbing agent level on the reflectivity of the coating.

Figure 7 shows the effect of the absorbent amount on the electromagnetic wave frequency for each specimen. From this figure, it is clear that different absorbent amounts clearly lead to significant variations in the composite absorption range and intensity. More specifically, Figure 7 and Table 5 show the results for a BaFe_12_O_19_-PPy composite with an absorption layer thickness of 2.5 mm wherein the minimum values of the reflection losses (reflection peaks) in the gigahertz frequency band are shown. The figure shows that in the 8–18 GHz frequency band, a 5% absorption level led to a reflection loss of −9.8 dB for the BaFe_12_O_19_-PPy composite. In addition, the minimum reflection loss of −14.21 dB was reached for this sample with an effective bandwidth of 1.71 GHz. Furthermore, the reflection loss obtained for the BaFe_12_O_19_-PPy composite containing 7% absorbent was determined to be −10.0 dB, the minimum was observed at −14.27 dB, and the effective bandwidth was 1.88 GHz.

Finally, the results presented above clearly indicate that the BaFe_12_O_19_-PPy composite exhibits a lower reflection loss peak and a larger effective bandwidth at the X band when the thickness of the coating and the proportion of the absorbent are 2.5 mm and 5–7%, respectively, at which point the optimal absorption performance is reached, representing a reflection loss of 8.88–10.92 GHz below −10 dB. The microwave absorption of BaFe_12_O_19_/PPy reaches the same microwave absorption capabilityas was found inearlier research [15].

## 4. Conclusions

Electromagnetic functionalized core–shell BaFe_12_O_19_-PPy microstructured composites were synthesized via an in situ chemical oxidative polymerization approach using BaFe_12_O_19_ as a nucleation center based on the orthogonal experiment method. Characterization by Fourier-transform infrared spectroscopy and X-ray diffraction confirmed that there were no obvious chemical interactions between the BaFe_12_O_19_ core and the PPy shell. In addition, scanning electron microscopy imaging confirmed that nanocomposites with a core–shell structure were obtained, wherein the magnetic core consisted of ferrite and the conducting shell of PPy. The results showed that the size of BaFe_12_O_19_/PPy microstructured compositesis about 80nm. The cured coating with BaFe_12_O_19_/PPy composite has lower reflection loss peak and larger effective bandwidth at the X band when the proportion of the absorbent sample is 5%–7%, when the absorption performance is the best. The reflection loss is in the range of 8.88–10.92 GHz below −10 dB, and the coatings have good hardness, adhesion and flexibility. Moreover, the potential application of this composite was examined by testing its coating properties using an aluminum alloy substrate. The desirable hardness, adhesion flexibility, weather resistance, and wave absorption properties exhibited by this system indicate its potential for use in protection against electromagnetic irradiation, in microwave darkrooms, and in the electronic information technology industry.

## Figures and Tables

**Table 1:** Composition of the UV curing coating.

Material	Mass Ratio (%)
PUA(B-369)	10−19
EA (B-153)	26
TPGDA	40
TMPTA	10
1173	4
BaFe_12_O_19_-PPy	1–10

**Table 2:** Properties of the composite coating.

Test Item	Testing Result
Curing time Impact resistance Adhesion testing Hardness test Acid resistance Water resistance Oil resistance	50 s 48 cm Level 1 4H NC NC NC

**Table 3:** Electrical properties of the composites.

	EXPNO	A	B	C	D
Time/h	APS/g	SDBS/g	BaF/g	Electrical Conductivity	S/m
1	6	0.5	1.75	3.35	1.005
2	6	1	3.48	1.68	2.751
3	6	2	7	0.84	2.938
4	12	0.5	3.48	0.84	2.166
5	12	1	7	3.35	3.137
6	12	2	1.75	1.68	2.151
7	24	0.5	7	1.68	2.117
8	24	1	1.75	0.84	6.971
9	24	2	3.48	3.35	2.462
10	24	1	1.75	0	7.245

**Table 4:** Data analysis for the various experimental factors.

T ^[1]^	A	B	C	D	S
T1	6.693	5.289	10.128	6.603	2.069
T2	7.455	12.858	7.38	7.02	2.739
T3	11.55	7.511	8.373	12.651	2.468
t1 ^[2]^	2.231	1.763	3.376	2.201	0.6896
t2	2.485	4.286	2.460	2.340	0.9289
t3	3.85	2.517	2.791	4.025	0.7528
R ^[3]^	1.619	2.523	0.916	1.824	0.6850

^[1]^ T is the sum of the factor test results, e.g., T1 = 1.005 + 2.751 + 2.938 = 6.693. ^[2]^ t is the mean value of the sum of the experimental results, e.g., $t1=\frac{6.693}{3}=2.231$. ^[3]^ R is the maximum–minimum of the t values. From the above two tables: R (B) > R (D) > R (A) > R (C).

**Table 5:** Absorption properties of the composites.

Absorbent Loading (%)	R Lmin/dB	Mix-Bandwidth below −10dB/GHz
1	-	-
3	−13.23	-
5	−14.27	1.71 (9.21–10.92
7	−14.21	1.88 (8.88–10.76)
10	−13.64	-

## Data Availability

Data of the present study are available in the article.
